# Method for converting CWEEDs weather files to EPW format for multiyear simulation of building thermal dynamics

**DOI:** 10.1016/j.mex.2020.101016

**Published:** 2020-07-29

**Authors:** Chun Yin Siu, Zaiyi Liao

**Affiliations:** a Department of Architectural Science, Ryerson University, Canada; b College of Hydraulic and Environmental Engineering, China Three Gorges University, China

**Keywords:** Building Energy Simulation, Historical Weather Files, Weather File Conversion

## Abstract

This paper outlines a method in converting Historical CWEEDs (Canadian Weather Energy and Engineering Datasets) stored in WY3 format into individual weather files in EPW format so it could be used for Building Energy Simulation (BES). The method outlined is independent of the programming language used, however, a sample MATLAB script is presented to illustrate its feasibility. Highlights of the methodology are as follow:

• Key feature in weather converters created under this methodology is the ability to divide multiyear weather data stored in WY3 format (with. wy3 extension) into individual years in EPW format (Standard mode)
• And provide ability to extract weather data within a defined time range

## Graphical abstract

**Figure:**
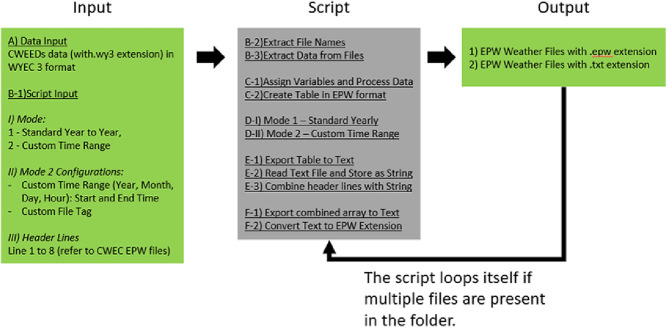

**Specifications Table**

**Table:** 

Subject Area:	Energy
More specific subject area:	*Building Energy Simulation*
Method name:	*Method for Converting Weather Files from WYEC3 format to EPW format*
Name and reference of original method:	
Resource availability:	*Historical Weather Data in WY3 format is needed as input data. This method is specific to Canadian Weather Engineering and Energy Datasets, which can be acquired from the Environment and Climate Change Canada weather database*[1]*.*

## Method details

This paper outlines a methodology to convert CWEEDs (Canadian Weather Energy and Engineering Datasets) historical weather data stored in WYEC3 format into the more commonly used EPW (Energy Plus Weather File) weather file format. The purpose of this method is to create script based weather converters for processing large quantities of weather data – in particular the .wy3 files released by Environment and Climate Change Canada [1] that contains multiyear weather data. One of the main features that differentiates this method is its inclusion of ability to create weather files with custom multi-year time range.

CWEEDs is a set of historical weather data released by Environment and Climate Change Canada accompanying the release of CWEC (Canadian Weather Year for Energy Calculation) Typical Year weather files [1]. Typical Year weather files such as CWEC uses statistical methods to select 12 representative months of hourly weather data to represent the long term median weather condition of a location (usually weather stations); whereas historical weather files such as CWEEDS contains all hourly weather data within a certain time range and serves as data source for Typical Year weather file assembly [2]. While the CWEC weather files are released in EPW weather file format – a weather data format created for Energy Plus which is compatible with most commercial Building Energy Simulation packages, CWEEDs are only currently available in WC3 format [2]. WY3 weather data format has a fixed width data structure which is typically more laborious to retrieve data than the more common csv type data format. There are already existing weather file conversion tools that can convert various forms of weather data into EPW format, including one that is developed by Energy Plus [3]. The Energy Plus weather data converter can be used to convert files in WYEC 2 (Weather Year for Energy Calculation 2) format which is used in previous CWEEDS and CWEC releases in the 1990s and 2005, but not WY3 which is used in the newer release of CWEEDS and CWEC in 2016. User defined weather data stored in csv spreadsheets with EPW format can also be compiled using the Energy Plus weather data conversion tool [3]. The technical document accompanying the release of CWEC and CWEEDs [2] also indicated that there exists a script created by Crawley which converts WY3 files into EPW weather files – which is used in converting CWEC WY3 files to EPW. It is unclear about the functionality and availability of Crawley's script as it is not uploaded with the weather files, and to the best knowledge of the authors, the tool is currently not released for public use. As for the other mentioned tools, they are focused on creating single year weather files, in which handling large amounts of multiyear weather data can be laborious and prone to inconsistency.

The structure of this paper is as follow – First, the general stages of the methodology that is independent of programming language used is summarized; and then a sample script created for MATLAB is presented.

The following is a brief outline of the general stages of the methodology:

1) Obtain data
2) Import data
3) Data processing and organization
4) Packaging weather data into appropriate timeframes
5) Adding header lines
6) Export data into proper file extension

### Obtain data

The original data – CWEEDs (Canadian Weather Energy and Engineering Datasets) historical weather data is obtained from the Environment and Climate Change Canada web database [1]. The database contains both Historical and Typical weather data for 492 locations across Canada [2]. This methodology and the accompanied sample script work with CWEEDs weather data organized in the WY3 file format.

### Import data

First, the weather data obtained from the CWEEDs weather database has to be imported. To read the CWEEDs weather data contained in files with .wy3 extension, the file has to be first converted into a text file with .txt extension, this enables the text file based importing functions in different programming languages to recognize the file. Since the original CWEEDs data are organized in a fixed width format, familiarity of the location of data is critical. To clarify, fixed width format which relies on the precise length and location of data entry is different from the more commonly used comma separated formats used in EPW weather files, where individual data entries are separated with commas. In general, comma separated format is more widely used due to its flexibility in data organization in terms of length and its relative location. The proper location and format of the CWEEDs weather data is documented in detail in the technical report [2] accompanied the CWEC and CWEEDs weather file update in 2016.

### Data processing and organization

Since CWEEDs (in WY3 format) and EPW weather files are organized in different data formats, some of the fields in CWEEDs cannot be directly used in EPW files without processing. Table 1 summarizes the organization of the two weather files and processing work that needs to be completed. Processing work required can be summarized under 3 categories – conversion, estimation and filling in missing flags.

**Table 1:** Comparison of weather elements in EPW and WY3.

Elements in EPW {units} [5]	Equivalent Elements in CWEEDs WY3 {units} [2]	Processing Completed	Used in Energy Plus [5]
Date - Year	Date - Year		Yes
Date - Month	Date - Month		Yes
Date - Day	Date - Day		Yes
Time -Hour	Time -Hour		Yes
Time- Minute		Minutes are set to 0 since all recordings are at the hour	
Data Source and Uncertainty Flag		Used the generic Source flag from CWEC 2016 found in [1]	
Dry bulb temperature{C}	Dry bulb temperature {0.1°C}	Conversion to°C – conversion factor: 0.1	Yes
Dew point temperature{C}	Dew point temperature {0.1°C}	Conversion to °C – conversion factor: 0.1	Yes
Relative Humidity {%}		Calculated based on psychrometric relationships found in [4]	Yes
Atmospheric Pressure {Pa}	Station pressure {10 Pa}	Conversion to Pa – Conversion factor: 10	Yes
Extraterrestrial Horizontal Radiation {Wh/m^2}	Extraterrestrial irradiance {kJ/m2}	Conversion from irradiance to radiation – conversion factor: 1/3.6	No
Extraterrestrial Direct Radiation {Wh/m^2}		Conversion from irradiance to radiation – conversion factor: 1/3.6	No
Horizontal Infrared Radiation Intensity {Wh/m^2}		Missing flag is used to trigger estimation in Energy Plus as indicated in [5]	Yes
Global Horizontal Radiation {Wh/m^2}	Global horizontal irradiance {kJ/m2}	Conversion from irradiance to radiation – conversion factor: 1/3.6	No
Direct Normal Radiation {Wh/m^2}	Direct normal irradiance {kJ/m2}	Conversion from irradiance to radiation – conversion factor: 1/3.6	Yes
Diffuse Horizontal Radiation {Wh/m^2}	Diffuse horizontal irradiance {kJ/m2}	Conversion from irradiance to radiation – conversion factor: 1/3.6	Yes
Global Horizontal Illuminance {lux}	Global horizontal illuminance {100 lux}	Conversion to lux – conversion factor: 100	No
Direct Normal Illuminance {lux}	Direct normal illuminance {100 lux}	Conversion to lux – conversion factor: 100	No
Diffuse Horizontal Illuminance {lux}	Diffuse horizontal illuminance {100 lux}	Conversion to lux – conversion factor: 100	No
Zenith Luminance {Cd/m2}	Zenith luminance {100 Cd/m2}	Conversion to Cd/m^2 conversion factor: 100	No
Wind Direction {Degrees}	Wind direction {0‐359 degrees}		Yes
Wind Speed {m/s}	Wind speed {0.1 m/s}	Conversion to m/s – conversion factor: 0.1	Yes
Total Sky Cover {0 to 10}	Total sky cover {0‐10 in tenths}		Yes
Opaque Sky Cover {0 to 10}	Opaque sky cover {0‐10 in tenths}		Yes
Visibility {km}	Visibility {100 m}	Conversion to km – conversion factor:0.1	No
Ceiling Height {m}	Ceiling height {10 m}	Conversion to m– conversion factor:10	No
Present Weather Observation	N/A	Missing Flag is used, refer to reference [5] for specific flag	Yes
Present Weather Codes	Present Weather *not used if previous field is 9		Yes
Precipitable Water {mm}		Missing Flag is used, refer to reference [5] for specific flag	No
Aerosol Optical Depth {thousandths}		Missing Flag is used, refer to reference [5] for specific flag	No
Snow Depth {cm}	Snow Cover	The original CWEC snow depth records only indicate snow coverage as snow cover (1) or no snow cover (0) [2]. In EPW weather files, snow cover should be a measurement of depth [5]. Upon inspecting convention used in CWEC, it is found that snow coverage is used directly in the EPW version, therefore conversion is not completed	Yes
Days Since Last Snowfall		Missing Flag is used, refer to reference [5] for specific flag	No
Albedo		Missing Flag is used, refer to reference [5] for specific flag	No
Liquid Precipitation Depth{mm}		Missing Flag is used, refer to reference [5] for specific flag	Yes
Liquid Precipitation Quantity {hr}		Missing Flag is used, refer to reference [5] for specific flag	No

Conversion is completed for fields that are not in the appropriate units. While most conversions are straight forward, for clarity, Table 1 also shows the conversion factors used.

Estimations are completed for fields that could be estimated based on the recordings in CWEEDs data. For instance, relative humidity is not directly recorded in CWEEDs weather data and therefore is estimated based on psychrometric relationships as outlined in [4].

Last but not least, fields that are missing are placed with the appropriate missing flags as stated in [5]. When missing flags are placed in the weather file, and the field is required for energy calculation, “appropriate” values are used in its place [6]. National Renewable Energy Laboratory [6] outlines the default values used in Energy Plus when missing flags are indicated in the weather files. It also worth noting that not all fields in the EPW weather files are used during Energy Plus simulations, Big Ladder Software [5] states clearly fields that are not used during simulation and the information is also summarized as part of Table 1.

The extracted fields from WY3 files are then organized in a table in EPW format. The EPW file format can be found in [5], or it can be easily referenced by converting a CWEC Typical Year weather file (in EPW format) into a comma separated file using the Energy Plus weather file converter [5]. By doing so, the converter also adds two rows of labels that clearly indicates the name of variable and units of each column.

### Packaging weather data into appropriate timeframes

One of the highlighted features of this methodology is the inclusion of custom time range packaging. The most conventional weather data organizational method for energy simulation is to separate weather files in years, since standard practice is to simulate singular typical years. Some simulation packages (such as Energy Plus) do accept multi-year weather input, however, creating weather files with custom time range can be labor intensive and prone to error/inconsistency, especially if it is created by copy and pasting raw weather data. This method, specific to CWEEDs historical weather data, has the ability to extract weather data in a specified time range directly from the datasets.

### Adding header lines

The required information and format of header lines needed for header lines in EPW weather files are documented in [5]. This is one of the features of EPW weather files, in which information such as design-day data and ground temperature data are included in the header lines. The design-day data can be obtained from the ASHRAE Handbook of Fundamentals [7]. The simplest way to include the header lines is by using the header lines of an existing EPW weather file created for the same location. In this case, each of the CWEEDs location has an accompanied CWEC Typical Year weather file, and the header lines are can be added to the custom EPW weather files.

### Exporting data into proper file extensions

This final step is rather simple and self-explanatory, the goal is to export the weather file in the correct file extension so the simulation package can recognize the file.

## Example code (MATLAB)

To illustrate the methodology, a sample script is written using MATLAB 2019 [8]. The following section begins with an overview of how the script could be used to convert WY3 files into EPW files; and then a more thorough explanation on the key components and logic of the script is presented. In addition, the full script with comments is included as a supplementary file.

### Overview of how the script works

**Fig. 1:**
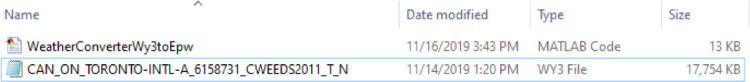
Sample Folder with Script and Weather File to be converted.

**Fig. 2:**
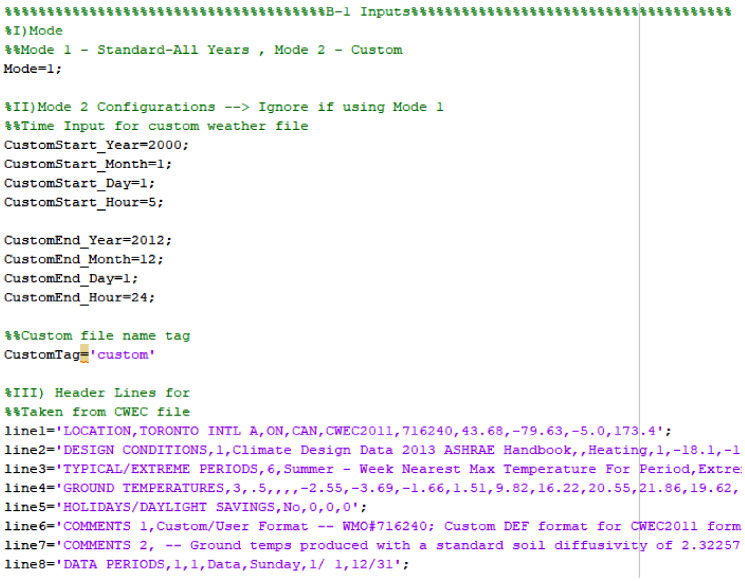
User Input Parameters.

**Fig. 3:**
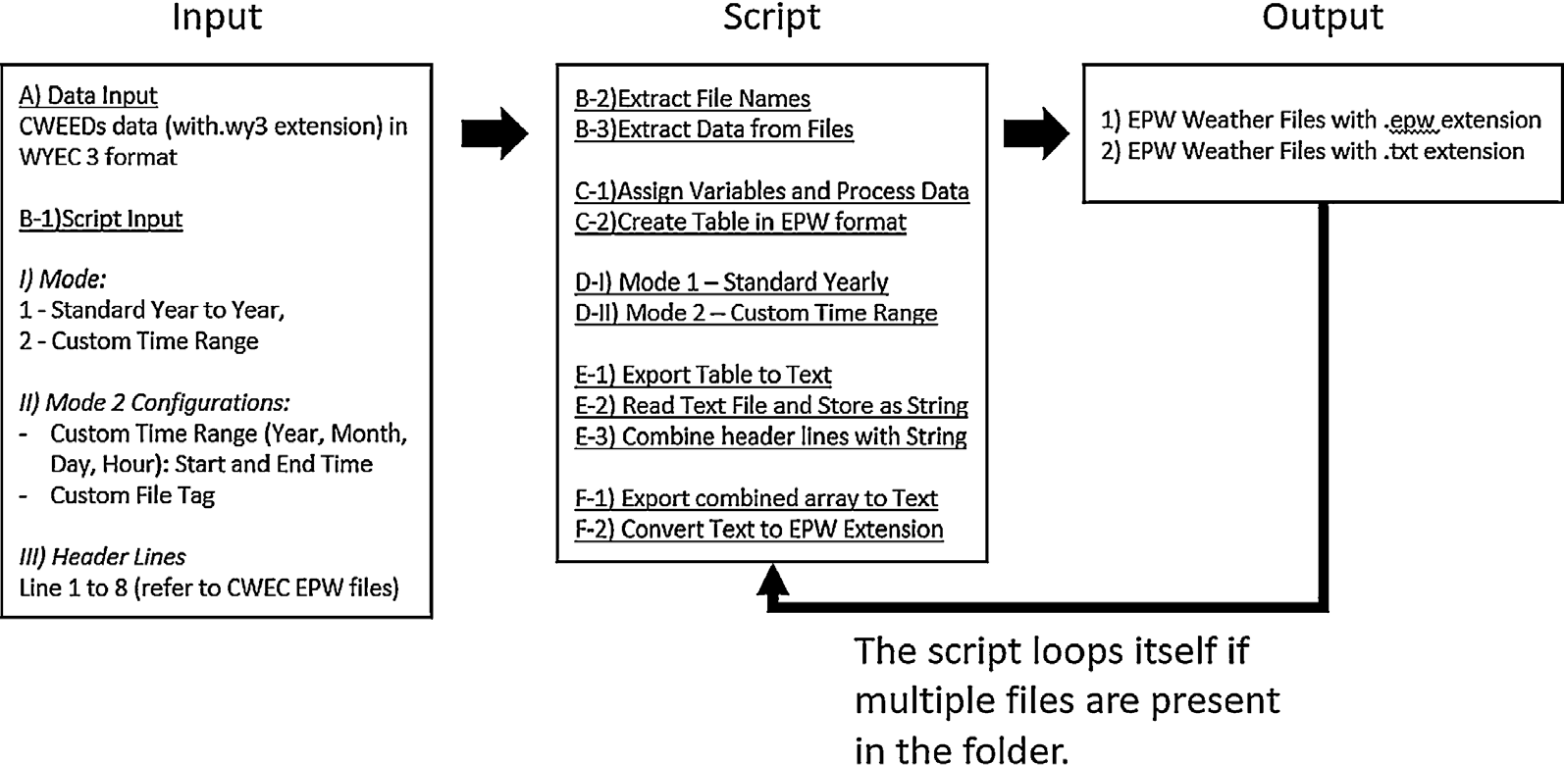
Overview of MATLAB script.

The script is designed so it could be used easily by users with some programming experience, the required input parameters are located at the beginning of the script with clear labels. Fig. 1 is an example of how an input folder should be set up. Fig. 2 is an example of the input parameters. Fig. 3 shows an overview of the script. The following are steps to run the script:

1) Obtain data from the online database, and place in a desired output folder.
2) Place script in a folder with all the .wy3 files that needs to be converted.
3) Open script with MATLAB
4) Configure Input parameters
5) Run the script
6) Double check the output files

### Overview of script

#### Obtaining data (Not Part of the Script)

This part is not being completed by the script. Users should obtain files from [1] and place it in a folder where the script is located (see Fig. 1).

#### Import data

*B-1) Script Input*

This is located at the beginning of the script (see Fig. 2). The first input is mode selection (B-1-I) – mode 1 results in standard output, where all historical data is separated into individual years; mode 2 results in custom output, where only weather data from the selected time range (in B-1-II) will be extracted to the output weather file. Users can also insert a custom file tag (in B-1-II). Lastly, the header lines can be added in B-1-III.

B-2) *Extract File names*

This is to extract files names with .wy3 extension in the folder, so the script knows which files are being converted.

B-3) *Extract Data*

The script extracts data from the .wy3 files based on the fixed width location and stores them in a table named “T” so it could be used for further processing and reorganizing in the next step.

#### Data processing and organize data in proper format

*C-1) Assign Variables and Process Data*

For each relevant weather element, an array in the appropriate data type is created. As a rule of thumb, numbers are stored in “double” (numerical data type) arrays which allows for numerical operations and text are stored in “string” arrays.

*C-2) Create Table in EPW format*

All relevant weather elements are then organized into a new table that is structured following the EPW format.

#### Packaging weather data into the appropriate timeframes

An “IF” statement is used to control the two different modes – Standard Mode (Mode 1) and Custom Time Range Mode (Mode 2). Mode 1 is activated when mode=1 is indicated in the input section at the beginning of the script; whereas Mode 2 is activated when mode=2.

*D-I) Mode 1- Standard*

**Fig. 4:**
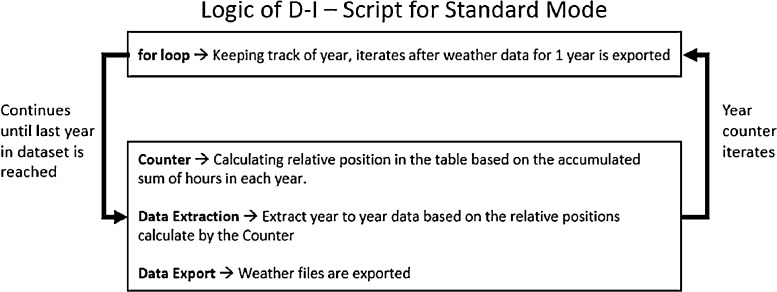
Logic of D-I - Script for Standard Mode.

As shown in Fig. 4, the logic of standard mode is simple – a “for” loop counter is created to keep track of years. The for loop begins in the first year (year column entry for the first row of data); and ends after the last year (year column entry for the last row of data). Within the for loop there are 3 components:1) a hour position counter that keeps track of the relative start and end time of each year(in hours);2) a data extraction component to extract data based on the relative start and end time; and 3) data export components housing scripts in part E and F.

D-II) Mode 2- Custom Time Range

**Fig. 5:**
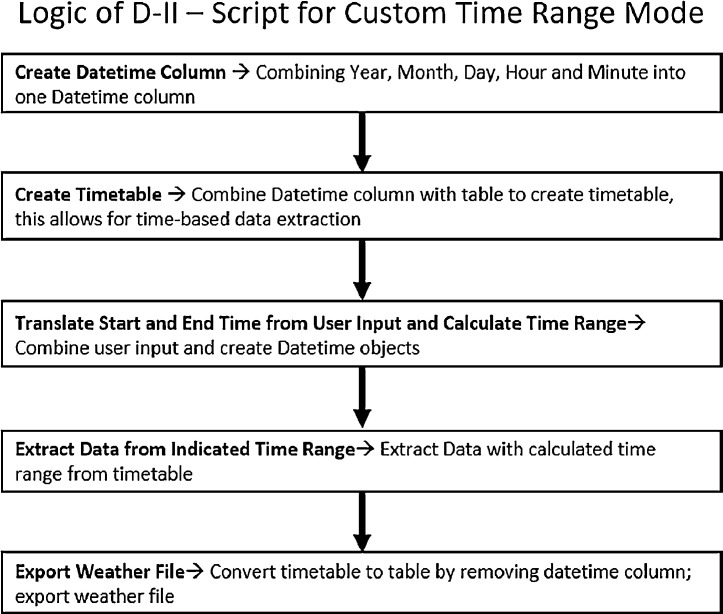
Logic of D-II - Script for Custom Time Range Mode.

As shown in Fig. 5, D-II consists of 5 components: 1) creation of Datetime Column – this is for the purpose of creating a Datetime index for the dataset;2) Create a Timetable – for the purpose of enabling Datetime indexing for the dataset; 3) Calculate time range based on user inputs; 4) extract data within the time range based on Datetime indexing; 5) weather files export component that houses script from parts E and F.

#### Adding header lines

This step is straight forward logic wise, where the goal is to add header lines indicated in user input. However, in execution, multiple steps are required due to different data storage dimensions and structure. The greatest issue is the number of columns in the weather data table is not the same as the number of columns used to store the header lines, therefore they cannot be concatenate directly. A workaround is to store the data table in a singular string array, so the headlines can concatenate vertically.

E-1) Export Table to Text

First is to export table into a text file, this is for the preparation of part E-2. During this process, the header line from the table is also removed.

E-2) Read Text and Store as String

Once the table is stored as a text file, it can be read stored as a single string.

E-3) Combine Header lines with String

The header lines (line 1 to 8) are then combined with the string that contains all the data.

#### Exporting data into proper file extensions

The data export is completed in 2 steps – export to txt then conversion to epw extension. This part of the script will not be discussed in detail, since the logic is straight forward.

F-1) Export Combined Array to Text

F-2) Convert Text to EPW Extension

## Appendix Supplementary materials

Image, application 1

## References

[1] Environment and Climate Change Canada, “Engineering climate datasets,” 2019. [Online]. Available: http://climate.weather.gc.ca/prods_servs/engineering_e.html.

[2] Morris R. (2016). Final Report – Updating CWEEDS Weather Files.

[3] Big Ladder Software, “Using the weather converter,” *Auxiliary Programs — EnergyPlus 8.3*. [Online]. Available: https://bigladdersoftware.com/epx/docs/8-3/auxiliary-programs/using-the-weather-converter.html.

[4] Bureau of Meteorology -Australian Government, “Calculation of relative humidity.” [Online]. Available: http://www.bom.gov.au/climate/averages/climatology/relhum/calc-rh.pdf.

[5] Big Ladder Software, “EnergyPlus weather file (EPW) data dictionary.” [Online]. Available: https://bigladdersoftware.com/epx/docs/8-3/auxiliary-programs/energyplus-weather-file-epw-data-dictionary.html.

[6] National Renewable Energy Laboratory, “Weather data – missing weather file data.” [Online]. Available: https://www.energyplus.net/sites/default/files/docs/site_v8.3.0/InputOutputReference/07-WeatherData/index.html#missing-weather-file-data.

[7] ASHRAE (2013). 2013 ASHRAE Handbook: Fundamentals.

[8] “MATLAB Release2019a.” The MathWorks Inc, Natick, MA, United States.

